# A qualitative systematic review on the experiences of homelessness among older adults

**DOI:** 10.1186/s12877-022-02978-9

**Published:** 2022-04-25

**Authors:** Phuntsho Om, Lisa Whitehead, Caroline Vafeas, Amanda Towell-Barnard

**Affiliations:** 1 School of Nursing and Midwifery, Edith Cowan University, Building 21, Level 4, PhD room, Joondalup Campus, Joondalup, Western Australia Australia; 2 Faculty of Nursing and Public Health, Khesar Gyalpo University of Medical Science of Bhutan, Thimphu, Bhutan; 3 The Centre for Evidence-Informed Nursing, Midwifery and Healthcare Practice, a JBI Affiliated Group, Joondalup, Australia

**Keywords:** Homelessness, Older adults, Ageing, Health and wellbeing, Experience, Needs, Qualitative, Systematic review

## Abstract

Adults who experience homelessness for an extended period of time also experience accelerated ageing and other negative impacts on their general health and wellbeing. Homelessness amongst older adults is on the rise, yet there are few systematic reviews investigating their experiences. Thus, this review classifies and synthesises qualitative research findings of studies published between 1990 to 2020 that have examined the needs and challenges of homeless older adults to elucidate their journey of homelessness. Seven papers met the requirements for inclusion. Three main themes were identified in the review: - (1) Pathways to homelessness, (2) Impact of homelessness, and (3) Outcomes and resolutions. This review collates current evidence on what is known about the experience of homelessness among older adults. In this study, homeless older adults identified a wide range of challenges associated with the experience of homelessness.

**Supplementary Information:**

The online version contains supplementary material available at 10.1186/s12877-022-02978-9.

## Background

The population globally is ageing. Although, ageing is truly a triumph of development, this demographic change presents both advantages and challenges. The concept of successful ageing is to “add life to years” rather than adding days to life and is about maximizing wellbeing and happiness for the older adult [1]. The risk of developing physical and mental health issues among older adults along with associated costs are linked to a higher demand for health and social care [2].

Theories on ageing have been developed with the goal of understanding the ageing process and how best to support “healthy ageing at home” and “ageing-in-place” [3] however these do not consider older adults who do not live in a supportive environment or adults who are homeless. The home setting can be a place associated with poor subjective well-being and some older adults may feel compelled to leave the home setting as a result [4].

There is no consistent definition of homelessness, rather it has been confined to socio-historical, geographical, and cultural contexts from which the term is drawn [5]. Homelessness can be defined by a range of categories: absolute and or hidden with homelessness defined as sleeping in parked cars or parks, in emergency shelters, or in temporary shelters (couch surfing) with no or minimal health and safety requirement standards, and risk to personal safety [6–8]. This includes people residing in sub-standard housing such, as single-room occupancy hotels, or cheap boarding houses, as well as low-cost tiny, lodgings with minimal amenities [9, 10].

There is an increasing rise in homelessness among older adults and older homeless adults have been identified as the “new homeless”, a “forgotten group” and a “hidden group” [9, 11, 12].

The reasons for homelessness amongst older adults are diverse. These can include: the impact of natural disasters; the availability of affordable housing, including rising rental costs, a decline in social welfare and support programs; financial insecurity; a lack of social amenities; and increasing rates of mental health issues, combined with various addictions, including gambling [2, 9, 11–16]. In addition to this, family relationship breakdowns, or the death of loved ones, can cut people’s social connections, resulting in older adults experiencing homelessness for the first time. This displacement of older more vulnerable adults can lead to deprivation including the basic need for a place they can relate to as home, subsequently leaving them homeless [3, 7, 15, 17].

Molinari, Brown and Frahm et al. (2013) found homelessness was unanimously perceived as a humiliating experience by homeless older adults [13]. According to a survey conducted by the United States Department of Housing and Urban Development, over 15% of 634,000 homeless individuals were 50 years or older, where the number of homeless people aged over 65 has been projected to double by 2050 [13]. The same survey reported that in the United States alone, adults as young as 50 years of age were facing challenges of homelessness, effectively accelerating ageing processes. Further to this, homeless older adults face a greater threat of age-related disease burden, where they are more likely to experience: functional, auditory, visual, and neurological impairments, frailty, emotional distress, and urinary incontinence, at higher rates than in the general community [18].

Similarly, van Dongen et al. have reported within a longitudinal cohort study, that older homeless adults, unlike their younger counterparts, reported a higher incidence of cardiovascular disease and visual problems, as well as reporting limited social support from family and friends or acquaintances, and limited medical or hospital care use in the past [19].

However, there is limited published research identifying the distinct needs of homeless older adults. This is a critical gap in the literature, where a deeper understanding of the experiences of older adults who have been or are currently homeless is required.

### Aim

The main aim of this qualitative systematic review is to synthesise the evidence on the experience of homelessness of older adults.

## Methods

### Design

Using Joanna Briggs Institute (JBI) guidelines, a meta-synthesis of global qualitative evidence was undertaken. Studies with titles and abstracts that met the analysis goals were retrieved and chosen, based on inclusion and exclusion criteria. These studies were further appraised to evaluate methodological validity by analysing evidence relevant to viability, appropriateness, meaningfulness, and effectiveness [20]. Qualitative and mixed-method studies with ample qualitative data in their results sections to allow secondary data analysis met the inclusion criteria. The sample comprised of older adults aged between 45 and 80 years that had experienced homelessness for at least one period. The search was restricted to studies that were published in English and available in full-text form, where studies with participants below 45 years, older adults in housing facilities, and aged care residents were excluded.

### Search methods

**Fig. 1:**
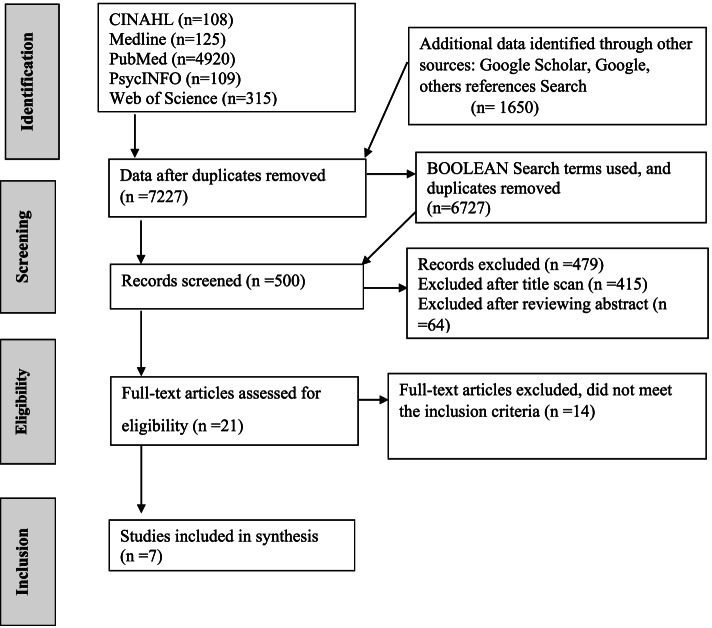
PRISMA flowchart

This analysis followed the Joanna Briggs Institute (JBI) method for systematic reviews [20]. A qualitative assessment and review instrument (JBI-QARI 10 item tool) [20] was used to facilitate the meta-synthesis. Results from the studies were extracted, categorised, and synthesised. Searches were conducted in PsycINFO, Web of Science, Google Scholar, Medline, PubMed, and CINAHL using appropriate search terms. Additionally, important citations were searched from reference lists of relevant articles. Searches were limited to published studies from 1990 to 2020 (see Fig. 1).

### Quality appraisal

Two reviewers independently assessed 21 articles for methodological quality in their design, conduct and analysis using the JBI-QARI 10 item tool [20]. Any discrepancies were discussed within the team. Out of the 21 articles, seven were included in the synthesis. Each selected study was re-read several times, discussed within the review team and data were abstracted for interpretation.

### Data abstraction

Findings relating to both current and past experiences of homelessness among older adults were extracted from the seven selected studies. A total of 56 findings were extracted. Each finding was reviewed and further compared and manually coded to identify themes. Table 1 lists the author and year, sample size, design, setting, and participant characteristics of the selected studies.

**Table 1:** Selected articles

Author and Year	Sample	design	City/Country	Participant Characteristics
Reynolds, Isaak, DeBoer, et al. (2016) [21]	*n* = 14	Qualitative research methods were guided by Grounded theory methodology	Vancouver, Winnipeg, Toronto, Montreal and Moncton / Canada	Homeless older people between ages 46–57 years.
Waldbrook (2015) [22]	*n* = 29	A mixed method study conducted using in-depth interviews within a theoretical constructivist paradigm	Toronto /Canada	16 male, 12 female, 1 transgender, ages between 45 and 65+ with a history of homelessness.
Grenier, Sussman and Barken, et al. (2016) [15]	*n* = 40	Mixed methods study, semi-structured interviews	USA	14 people with history of homelessness, 26 new to homelessness, aged between 46 and 76 years
Burns and Sussman (2019) [23]	*n* = 15	Constructivist grounded theory, in- depth interviews	USA	8 male, 7 female experiencing late-life homelessness, aged between 50 and 80
Viwatpanich (2015) [24]	*n* = 60	Mixed methods, in-depth interviews	Thailand	32 male, 28 female, Homeless, mean age 61.7 years
Molinari, Brown and Frahm et al. (2013) [13]	*n* = 45	Mixed methods, focus group and semi-structured interviews	USA	Homeless veterans aged between 49 and 72
Bazari, A., Patanwala, M., Kaplan et al. (2018) [25]	*n* = 20	Longitudinal study, qualitative, semi-structured interviews	USA	Homeless adults, aged 50+

#### Synthesis

Analysis of the seven reviewed articles was carried out using the qualitative evidence synthesis method [20] developed by JBI (2014). Qualitative findings from each study were first read and reread, followed by an identification of common themes. Recurring themes across studies were then grouped together in a meta-synthesis of the findings. This process comprised critical appraisal, data extraction, analysis, and a meta synthesis involving organisation and categorisation through decoding and encoding of the extracted data to produce a final summation of the findings. The qualitative evidence summation and synthesis were deliberated, cross-checked, and then reviewed by all the authors.

## Results

Of the seven studies identified for review (see Table 1 above), four studies directly explored pathways to homelessness amongst older adults. Individual study sample sizes ranged from 14 in Reynolds, et al. (2016) [21] to 60 in Viwatpanich (2015) [24]. Three studies applied in-depth face to face interviews, with three studies using semi-structured interviews, and one study conducting focus groups to collect data. The studies were conducted in three countries: Canada, USA, and Thailand.

**Fig. 2:**
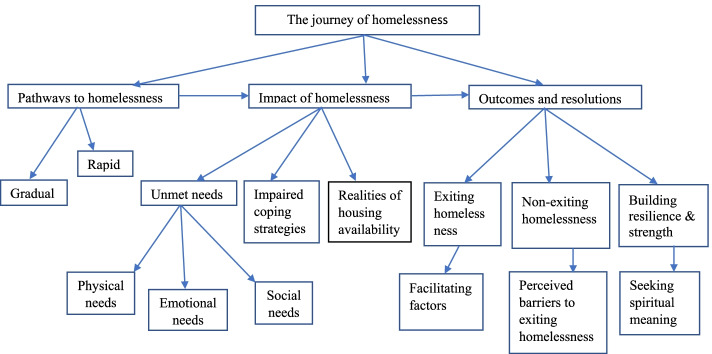
The Journey of Homelessness Model

Data synthesis commenced using open descriptive coding to search and identify concepts and finding relationship between them. Next using an interpretive process, the meaning units were categorised within each domain using labels close to the original language of the participants. The categorization of the data for each case was then followed by a cross-case analysis that examined the similarities and differences. Following categorisation, themes were conceptualised for each category. An overarching theme was identified: ‘the journey of homelessness’. Within this context, three core themes were identified: 1) Pathways to homelessness; 2) impact of homelessness; and 3) outcomes and resolutions, where each of these 3 themes had relevant sub-themes. (see Fig. 2).

The conceptual model depicted in Fig. 2 represents the overarching theme of the ‘journey to homelessness,” and key concepts and relationships between variables from the synthesis of the literature. Unlike other conceptual models that involve causal and directional relationships, this model is both directional and non-hierarchical. The model illustrates the pathways to homelessness, the associated impacts of homelessness and the outcomes of homelessness. The following section explores the three themes and sub-themes in more detail.

### Theme 1. Pathways to homelessness

The causes of homelessness were shown to be multifaceted, where pathways to homelessness revolved around a combination of individual, social, and structural factors. The reviewed data suggested that becoming homeless involved two distinct pathways: one that was gradual and one that was rapid.

#### Sub-theme 1.1: gradual pathway to homelessness

Findings from six studies contributed to this subtheme. This sub-theme captured the factors contributing to gradual pathways into homelessness amongst older people. These factors were identified as accelerated ageing, poverty, rising housing costs, failing and uncommitted social security systems, a lack of social programs and services, social distress, rural-urban migration, substance abuse and addiction, as well as estrangement from family or lack of living relatives [13, 14, 21–26].

The following quotations from these studies illustrate both estrangement from family and the impact of a lack of support from social services:

*Many conflicts we had at that time, we never talked … never talked in normal way … nothing clear between us, emotion never came clear...they did not want to talk to me, not even to look at my face … I could not stand it, I surrendered. Beating and scolding by descendants is not in our tradition, no respect, if they did not want me to stay with them, I moved out* [24]*.*

*I submitted applications for low-income housing, I’ve been on the waiting list, seven years is a long time, especially at my age* [22]*.*

Personal vulnerability to difficult familial relationships, neglected needs and unstable housing were the most cited causes of homelessness amongst these older adults [3, 9, 14, 25].

Two studies [15, 21] described a pathway to homelessness as related to alcoholism and drug abuse, as highlighted in the following quotation:

*I got into crack cocaine, I got into hooking, I got into anything you could think of I guess . . . So it was my addictions that brought me down, and unhealthy relationships* [25]*.*

Feeling ‘homeless at home’ [27] due to loneliness was noted by some older adults as their reason for ‘living on the streets’. For example, homeless older adults that experienced social rejection and conflicts with housing management, neighbours, and roommates, noted this to ultimately lead them to homelessness. For example, one participant stated, “I have lived alone and never really felt at home, because to me home is a place that includes other people, your family” [23].

#### Sub-theme 1.2: rapid pathway to homelessness

Some older adults described the process of homelessness as ‘rapid’. A rapid pathway to homelessness was associated with abrupt life changes such as losing a loved one, divorce, and the impact of these losses on their lives. The two quotes below highlight rapid pathway process:

*Losing them, let’s just say it evaporates over time. It’s the fact that I wake up like I am here that I can’t accept … homeless … in the street. I sold everything, every single thing! I never thought I’d end up like this. It’s like starting from zero* [23].

*I had a wife, then she died, I did not know where to go, what to do, I turned homeless* [24]*.*

Older adults that faced a series of losses and a rapid deprivation of social support systems noted the experience of disrupted circumstances. Accordingly, they noted their fear of losing their independence and ‘sense of self’ resulted in their resistance to any help that was offered, in turn contributing to their homelessness.

### Theme 2. Impact of homelessness

Findings from five studies contributed to this subtheme. Homelessness and ageing were presented to form a ‘double jeopardy’ where homelessness aggravated the challenges of old age [15, 21–24].

This theme included the subthemes of: unmet needs, coping strategies, and the realities of housing availability.

#### Sub-theme 2.1: unmet needs

‘Unmet needs’ amongst older homeless adults were categorised as involving physical, emotional and social needs leading to despair and destitution. As this quote below highlights:

*I’m supposed to get a pneumoscopy, but where am I, where do I stay? How can they get a hold of me? I don’t have money to get around* [15]*.*

##### Sub-theme 2.1.1: lack of physical wellbeing

Findings from six studies [14, 15, 18, 21, 22, 24] contributed to this subtheme. Physical decline and physical disability were described as exacerbated by the experience of being homeless. Participants described a relationship between age and frailty, fatigue, poor physical health, and impaired mobility while homeless, as these quotes demonstrate:

*Ah! Walking all day, for me, it’s very hard on the body, ok. Sleeping outside on a park bench, that’s very, very hard on the body. The bones, the humidity. Just leaving in the morning and then not going to work. … You’re always faced with the outdoors, and always faced with walking, walking. It’s not easy walking from downtown* [15]*.*

*My health was very poor. I was very prone to pneumonia. I was taken out of the shelter in the ambulance and it was later determined that I had actually contracted tuberculosis* [22]*.*

*At that time, I got Psoriasis, I knew that it was disgusting … . It looked scary. I am much too old. It is so difficult to find a job … nobody needed me … so I decided to stay and sleep here* [24]*.*

Homelessness in later life was shown to often be linked to a multitude of health problems. Most studies described older homeless people as living with physical health problems including chronic diseases such as hypertension, diabetes, bone and joint diseases, respiratory illness, and skin diseases [14, 21, 22]..

##### Sub-theme 2.1.2: lack of emotional wellbeing

Findings from five studies contributed to this subtheme. Accordingly, homelessness was described as contributing to poor emotional health related to social exclusion and isolation amongst older adults. Further, homelessness was associated with cognitive impairment, stigma, shame, stress and anxiety, as well as depression amongst homeless older adults [15, 21, 24, 25]. Homelessness was described as a humiliating and degrading experience, as evident in these quotes:

*At my age, I don’t see life ahead of me anymore. You see, I don’t know, I don’t see the end of the tunnel, … … It’s as if I wanted to erase myself* [15]*.*

*All I could think about was suicide. How did I end up here? When I think a lot to myself, what the hell am I doing?* [23]*.*

Feelings such as shame, demoralisation, and loss of dignity were described and these impacted on emotional health.

Opportunities to improve emotional wellbeing were rarely described, however one example stood out as an exception and this was related to volunteering:

*One thing I didn’t expect was when I helped people with whatever issues they were having on their bicycle, I really enjoyed that. It gave me a chance to teach someone* [25]*.*

Examples such as these were rare, with social exclusion and the lack of opportunity to contribute and connect with others more commonly described.

##### Sub-theme 2.1.3: lack of social relationships

Findings from four studies contributed to this subtheme. Social relationships were described as central to creating a life that had meaning and familial interactions. Disconnection from loved ones was associated with feelings of unhappiness [13, 15, 27], while companionship was shown to improve wellbeing [25]. Social relationships were shown to decline, leading to the experience of social exclusion and isolation.

*I am a walking dying woman. I walk until I can’t walk anymore, and then I sit. The busses pass me by. We are untouchables and I do not think anybody’s going to do anything about it* [25]*.*

*At my age, I don’t see life ahead of me anymore. Because everywhere I go: “Ah! He’s homeless.” It is as if I wanted to erase myself. I think that it’s more “society,” as such, that rejects homeless people* [15]*.*

*I think that living homeless, you exclude yourself, and a lot of other people exclude you. I was on the other side before becoming homeless. So, you know, the perception that people have, it plays a big part. … So that together makes it so that, if you don’t have family either, let’s say, you don’t have … close friends or a strong social network. Well, you experience all that, you live with loneliness and isolation* [15]*.*

#### Sub-theme 2.2: impaired coping strategies

Findings from four studies contributed to this subtheme. Older homeless adults described a range of factors as impacting their ability to cope. These included moving to shelters, challenges to adapt to their unique requirements, limited housing options, limited income supports, social exclusion, isolation, and a lack of coordination and access to community health and support services [13, 15, 23, 25].

As the quote below shows, there were expressions about the fear of homelessness and how long it will last:

*Struggling to get your basic needs met, scrounging, just trying to get by as best I can, and feeling desperation, humiliation, despair, a shocking feeling, full of fear, and turmoil. What’s tomorrow gonna bring? Why am I in this situation? How do I get out of it?* [13]

Coping with the harsh realities of homelessness in later life was described as being increasingly challenging for most older adults because older homeless individuals experience mental health disorders and acute or chronic physical illnesses.

#### Sub-theme 2.3: realities of housing availability

Findings from three studies [13, 15, 23] described the challenges experienced in accessing housing services and fulfilling requirements for safe, secure, and affordable housing. This theme captured impacts of poor coordination and communication between homeless veterans and housing intervention providers in regard to information for service availability, gaining access to homeless shelters and a lack of training and education by some housing providers especially with regard to homelessness.

*He … got this rule book and threw it at me. Find a place!* [13]

*You know, I’m 60, I’m not 20 anymore. So that’s what makes you tired, you get stressed. So, after that, they give you pills as a solution. I told the doctor, sorry I didn’t come here for pills, I came for housing* [23]*.*

*I submitted applications for low-income housing, I’ve been on the waiting list, seven years is a long time, especially at my age* [23]*.*

*I want a space where I can be well. I wasn’t well when I was young. I’ve never been well anywhere. I need a simple place … where I can have peace, and quiet … but not be all alone* [15]_*.*_

Older homeless adults described a need to create stability and escape homelessness through the provision of services, and in particular, housing. Older adults described how oscillating in and out of shelters prevented senses of safety, stability, or autonomy.

### Theme 3. Outcomes and resolutions

In four studies [13, 15, 21, 24] homeless older adults described how the outcomes and resolutions of homelessness involved overcoming both complex challenges and habituations. This theme encompassed the finding of directions and strengths to improve difficult situations and overcome challenges that occurred at the intersection of homelessness and ageing.

Three subthemes were identified within this theme: building resilience, strength, and hope; seeking spiritual meaning; and exiting the cycle of homelessness.

#### Sub-theme 3.1: exiting the cycle of homelessness

Some older adults moved out of the phase of homelessness and described facilitators and barriers to this transition whilst other described choosing to stay homeless until the end of their lives.

##### Sub-theme 3.1.1: factors facilitating the exit

Two studies [13, 15] contributed to this sub-theme, where older adults described means of overcoming challenges and establishing priorities in order to exit homelessness in later life. The results suggested that the creation of autonomy, flexibility, and privacy helped people feel belonging and often this meant living in a place where they could continue to drink and/or occasionally use drugs, have access to a health system to manage health problems; and have access to food and shelter facilitated exits.

*They listen to you and they help you with . . . your transition, your program. You sit down and you work the program out with them;” “If you have a question, you can walk in anytime and ask them what’s going on* [13]*.*

*In the next couple years, I hope to find myself an apartment for the few good years I have left, before the big pains of “aging” come* [15]*.*

Fulfilling financial support, housing and health care services was identified facilitate older adults exiting homelessness.

#### Sub-theme 3.2: remaining homeless

Some older adults experienced homelessness at a younger age and described continuing to be homeless in older age, where they oscillated between living in shelters and on the streets.

*I am used to being in this way, moved from place to place … me alone, without father and mother since childhood … it become normal and I feel happier, than to stay with others* [24]*.*

*It’s just a continual cycle. I just got sucked down into it, you know. It’s hard to describe because when I found myself there, I was just like, wow. How did I get here?* [21]

Participants described the chronic nature of homelessness as involving a challenge of disentangling themselves from the cycle of homelessness. A lack of tailored intervention programs to respond to homelessness in later life also prevented older adults from exiting homelessness.

##### Sub-theme 3.2.1: perceived barriers to exiting homelessness

In two studies [21, 24], older adults described experiences of vulnerabilities and challenges to exiting homelessness. Shelters were described as constraining and not being able to adapt to the unique needs of older adults. Where limited housing options were seen as available, income supports were described as limited, with a lack of coordinated and, accessible community health and social support services, impacting on participants’ ability to ‘feel in place’.

*My health pretty much stayed the same as when I was homeless. The conditions I have aren't gonna improve* [22]*.*

*It’s harder to keep a place, especially when you keep falling back in the same circle and you’re in the same crowd. I am finding out today, you keep falling back in the same circle, the same circle is not gonna change* [21]*.*

One participant described the difficulty of obtaining employment as a barrier to exiting homelessness:

*You know being 50 years old, it’s going to be really difficult to be able to reintegrate into the workforce* [21]*.*

Housing facilities and transition to housing shelters were shown to present challenges for homeless older adults. A lack of privacy, autonomy, rigid rules, and challenging interpersonal relationships within housing and shelter programs were identified as leading older adults to feel homeless at home.

#### Sub-theme 3.3: building resilience and strength

This sub-theme captured the life lessons, resilience, strength, and hope of older homeless adults, described as having formed through experiences and skills developed whilst living on the streets. This theme also suggests how individuals cope with difficult symptoms related to social support and, addiction, relying on positive things learned while living with other homeless people on the streets. Some older adults chose to stay homeless accepting homelessness as their fate.

*In the next couple years, I hope to find myself an apartment for the few good years I have left, before the big pains of “aging” come. I really want a normal life, get up in the morning, go to work, think about vacation. Hang out with other people … I don’t have a girlfriend but would like to start a life with someone else* [15]*.*

*What does ageing mean to you, getting older on the streets? A: Experience. Q: Ok. A: Wisdom. Q: Getting older on the streets, that’s how you see it, it’s the wisdom that you have gained. A: Yeah, that’s where I learned to be wise. Because there are several people who told me I am wise* [15]*.*

*I think because of karma … I accept it as punishment from bad deeds in my former life, but only in this life okay! Next life I am looking forward for a normal life, like others* [24]*.*

Most studies [3, 8, 13, 17] cited that wisdom, experience, and optimism were necessary in order to help older adults exit homelessness. Optimism instilled future hope and self-worth back into the self-esteem of homeless older adults.

##### Sub-theme 3.3.1: seeking spiritual meaning

In two studies [24, 25], older adults described finding meaning in life through adopting and accepting religious faith with a belief to achieve higher self-actualisation.

*I want to be closer to Dhamma (Buddhist teaching), I want to be a monk till I die* [24]*.*

*Meditate, just being by myself. Living the night, just being alone and listening to my music, that makes [my pain] feel better. I like jazz but I just listen to my music, just go away to myself. That makes me feel - I like being alone. I love being alone* [25]*.*

*When I feel [anger over my situation] I go to the water and I pray hard. I just start praising God until I can feel the spirit come over me to comfort me. I pray until He comes and allows his spirit to wrap his arm around me; I feel a lot better. A psychiatrist can’t tell me what’s wrong with me. For someone to try to help would mean a lot. I do not have nobody but to trust God. He’s my only psychiatrist* [25]*.*

Homeless older adults recognised and confirmed that psychosocial and existential symptoms caused as much distress as physical symptoms triggering negative changes in personality, energy, and motivation. Some homeless older adults viewed their age as a source of strength, wisdom, and experience in learning to manage their symptoms, describing themselves as survivors who had overcome significant hardships. Higher levels of wellbeing were likely to be achieved when older people sought spiritual meaning through religion, socialising, reading, meditating, volunteering, and introspection practices.

## Discussion

This review synthesised evidence generated from qualitative studies to provide a glimpse into the experiences of homeless older adults. The review has shown that while drivers related to entry into homelessness were diverse, two distinct trajectories underpinned the experience of becoming homeless amongst older adults. Older people that faced a sudden series of losses that completely overturned their circumstances fell into the ‘rapid pathway’ to homelessness. Participants on a ‘gradual pathway’ were shown to become homeless due to a range of factors, for example - addiction problems, physical and mental health issues, relationship break-ups, foster care, poverty, unemployment, and greater housing instability [13, 24]. Further to this, homeless older adults were shown to include a significant percentage of separated, divorced, or single individuals [28]. Likewise becoming single in later life was shown to be associated with homelessness amongst older people. Other studies found that ageing, its associated factors and a lack of stable housing were prominent reasons for homelessness [15, 22, 23].

Housing was perceived to offer a sense of security and a stable environment conducive for safe ageing. Further, housing was identified as offering protection from harsh weather and other dangers. Similar accounts relaying how the health of homeless older adults declined during episodes of homelessness was also reported [9]. Stable housing played an influencing role in physical health and general wellbeing. Although homeless older adults expressed satisfaction with life, they linked secure housing with healthy dietary habits, proper sleep patterns, enhanced self-care and reduced feelings of stress and anxiety [22]. In addition, this review found that most homeless older adults were more able to prioritise their health care needs when other necessities such as food and shelter were met. However, research has also suggested that living in scattered-site apartments can reinforce the process of social exclusion, and thus they are not appropriate for older adults living alone, with regard to their additional health and social needs [3, 10, 28].

Ageing intensified the adversities of homelessness experiences and presented a twofold risk where homelessness aggravated the challenges of old age and vice versa [15]. Old age and its associated conditions intensified older adults’ perceptions of homelessness later in life, including feelings of shame, anxiety, and worry. Studies by Cohen [9], Kwan, Lau and Cheung [29], and Molinari et al. [13], have unanimously shown older adults to perceive homelessness as a dehumanising experience. Homelessness was described as: struggling “to get your basic needs met,” “scrounging, just trying to get by as best I can,” and feeling “desperation,” “humiliation,” “despair,” “a shocking feeling,” “full of dread, turmoil,” “what will tomorrow bring? why am I in this predicament and how can I get over it?” [13]. For most participants, homelessness was not a preferred option.

The limitations of this review include the predominance of data collected in North America which may reduce the generalisability of the findings. Another drawback is that it presents only a cursory review of issues related to gender, race, and ethnicity. Finally, the qualitative data analysis applied by the majority of studies here is subjective, where outcomes could be affected by authors’ personal biases.

Despite these limitations, the review has conceptualised two divergent pathways into homelessness in later life, as well as the impacts of homelessness, drawing attention to a greater understanding of homelessness experienced by older adults.

The review sought to provide insight into the needs of homeless older adults. Awareness of the complexities faced by homeless older adults need to be acknowledged if policy and research are to support the population and improve access to resources and support. The review has highlighted areas for future research to expand knowledge and understanding of the unique needs and challenges of homeless older adults.

## Conclusion

Synthesis of seven studies resulted in the identification of an overarching theme relating to the ‘journey of homelessness’ and three major themes, each with subthemes, to describe older adults’ experiences of homelessness. A broad range of diverse settings, cultures, and countries with a particular focus on homelessness in later life were included. The review has revealed homogeneity of experiences amongst homeless older adults, with the need for access to appropriate and affordable housing and adequate support systems.

The findings have identified pathways to homelessness require different prevention and support measures. People in the study who described a gradual pathway needed social support to address distress, which might have helped them avoid losing their homes. Those individuals with rapid pathways unanimously concluded that homelessness could have been avoided if independence and self-sufficiency were less regarded as a norm by society.

## Supplementary Information

**Additional file 1.**

## Data Availability

The authors declare that all data generated or analysed during this study are included in this published article.
