# Supplementary material for: A qualitative systematic review on the experiences of homelessness among older adults
Source: BMC Geriatr. 2022 Apr 25;22:363. doi: 10.1186/s12877-022-02978-9 (PMC9040287; doi:10.1186/s12877-022-02978-9)
Supplement: Supplementary file 1 — Additional file 1. [file 12877_2022_2978_MOESM1_ESM.docx]

Appendix I: Appraisal instruments

QARI appraisal instrument

JBI QARI critical appraisal checklist for systematic reviews and research synthesis

Reviewer ______________________________________ Date_______________________________

Author_______________________________________ Year_________ Record Number_________

|  | Yes | No | Unclear | Not applicable |
| --- | --- | --- | --- | --- |
| 1. Is the review question clearly and explicitly stated? | □ | □ | □ | □ |
| 1. Were the inclusion criteria appropriate for the review question? | □ | □ | □ | □ |
| 1. Was the search strategy appropriate? | □ | □ | □ | □ |
| 1. Were the sources and resources used to search for studies adequate? | □ | □ | □ | □ |
| 1. Were the criteria for appraising studies appropriate? | □ | □ | □ | □ |
| 1. Was critical appraisal conducted by two or more reviewers independently? | □ | □ | □ | □ |
| 1. Were there methods to minimize errors in data extraction? | □ | □ | □ | □ |
| 1. Were the methods used to combine studies appropriate? | □ | □ | □ | □ |
| 1. Was the likelihood of publication bias assessed? | □ | □ | □ | □ |
| 1. Were recommendations for policy and/or practice supported by the reported data? | □ | □ | □ | □ |
| 1. Were the specific directives for new research appropriate? | □ | □ | □ | □ |

Overall appraisal: Include □ Exclude □ Seek further info □

Comments (Including reason for exclusion)

______________________________________________________________________________________________________________________________________________________________________________________

Appendix II: Data extraction instruments

QARI data extraction instrument

JBI QARI Data Extraction Form for Systematic Reviews


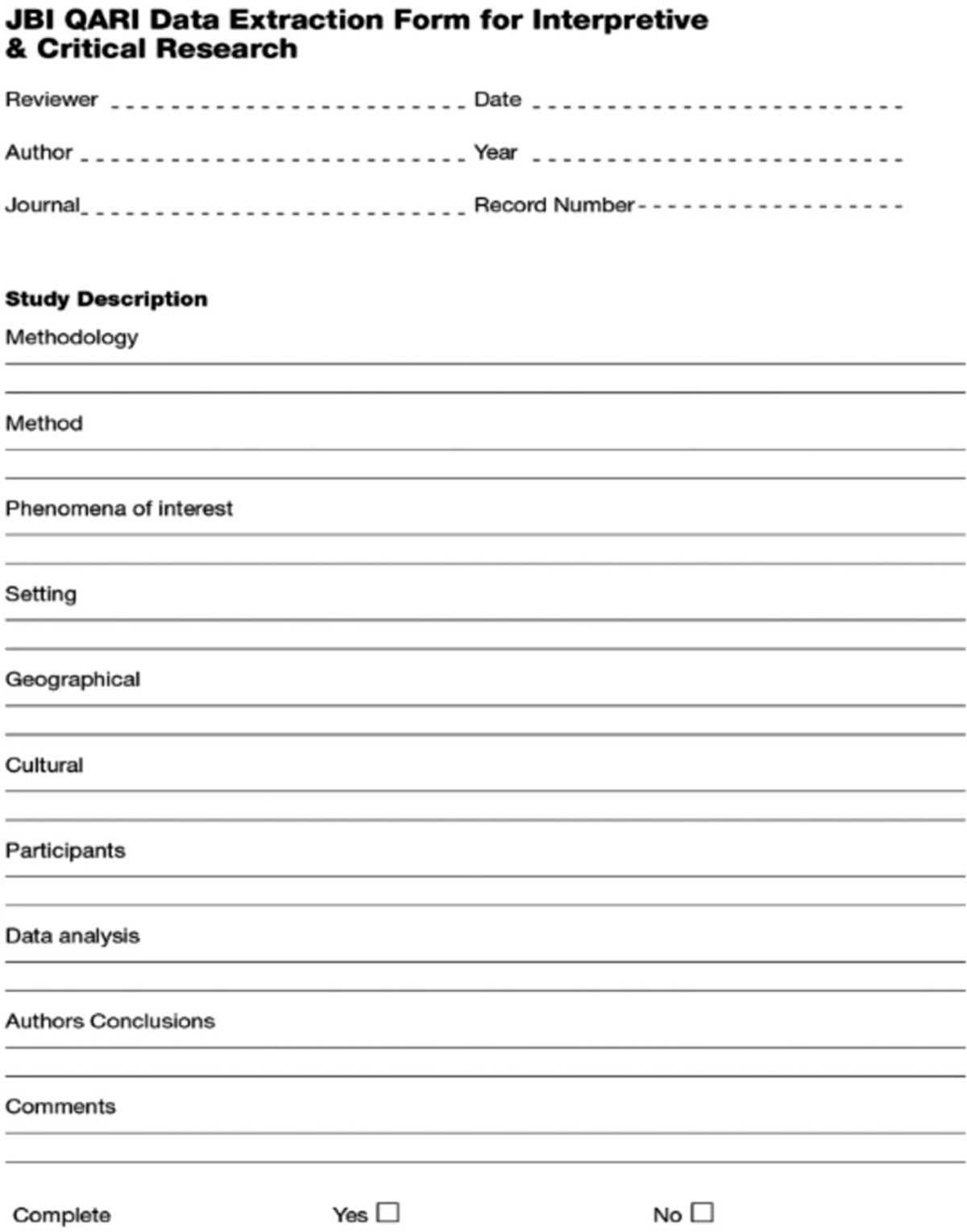


**
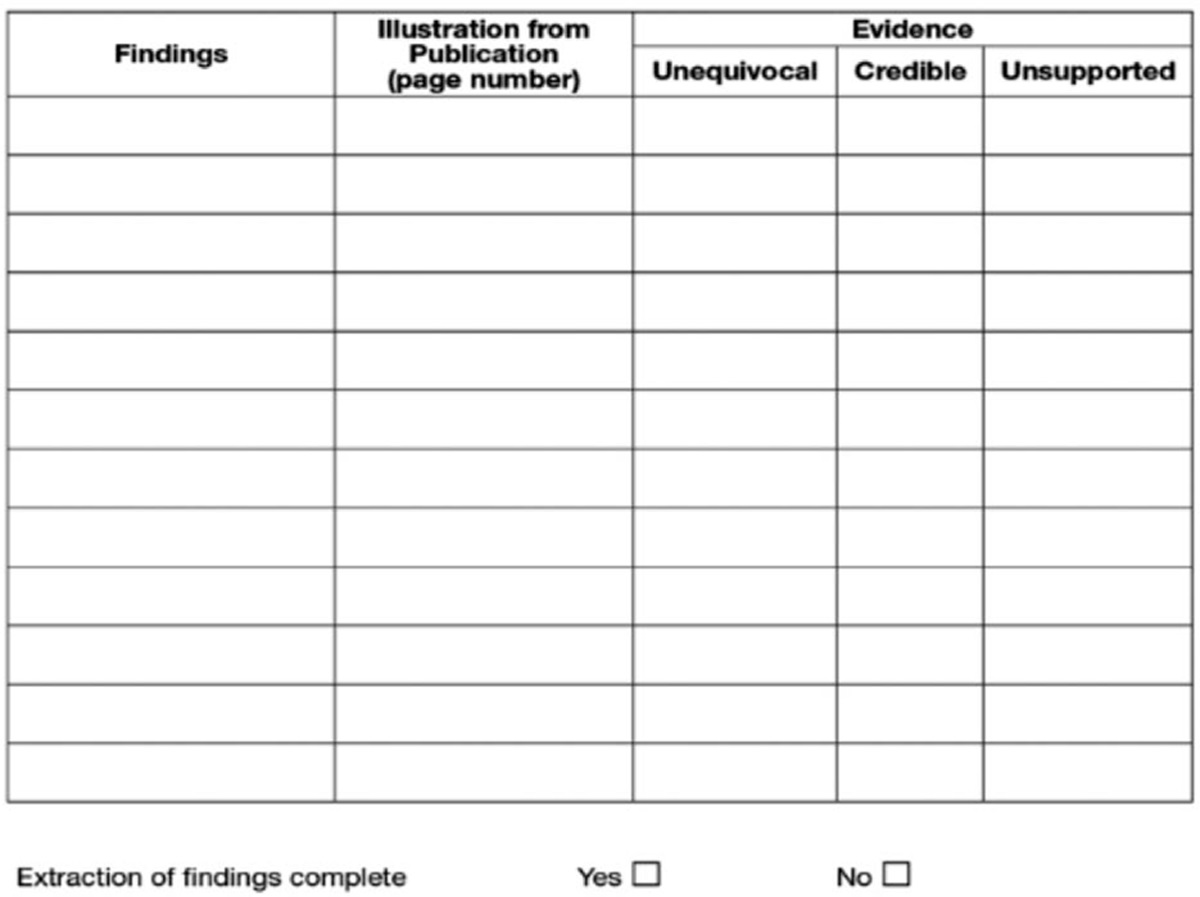
**
